# Early and late results of carotid endarterectomy: retrospective study of 70 operations

**DOI:** 10.1590/S1516-31802001000600005

**Published:** 2001-11-01

**Authors:** Eduardo Toledo de Aguiar, Alex Lederman, Celso Higutchi, Gerd Schreen

**Keywords:** Carotid artery, Endarterectomy, Arteries, Atherosclerosis, Cerebrovascular disease, Artéria carótida, Artérias, Aterosclerose, Endarterectomia, Doença cérebro-vascular

## Abstract

**CONTEXT:**

Indications and results of carotid endarterectomy have been defined from clinical multicentric trials like the European Carotid Surgery Trialists, North-American Symptomatic Carotid Endarterectomy Trial and Asymptomatic Carotid Atherosclerosis Study. The patients included in these trials were highly selected, as were the surgeons performing the operations. Clinical practice is different but the same results should be achieved.

**OBJECTIVE:**

To study indications, technique, early and late results, and whether carotid endarterectomy has been performed in accordance with standards defined by multicentric trials.

**DESIGN:**

Retrospective case report study.

**SETTING:**

A tertiary care private hospital.

**PARTICIPANTS:**

57 patients, on whom 70 carotid endarterectomies were performed over a 10-year period. The median age was 66.4 ± 7.8 years;43 (75.4%)were male, 41 (71.9%)hypertensive, 36 (63.1%)current smokers and 24(21.0%)had diabetes. Bilateral carotid stenosis was present in 31 (54.3%) patients, peripheral arterial occlusions in 32(56.1%) and ischemic cardiopathy in 25(43.1%). All patients hadhad angiography and 41 (71.9%)had also had a duplex-scan of neck arteries. Cerebral imaging via computerized to mography scan or magnetic resonance imaging was obtained for 36 patients. Patients were followed up over a period of one to 122 months.

**MAIN MEASUREMENTS:**

early and late post-operative death, earlyand late post-operative stroke, and recurrence of atheroma plaque and symptoms relative to carotid stenosis.

**RESULTS:**

Therewas one post-operative death(1.4%) caused by myocardial infarction and two early strokes(2.8%): a total complication rate of 4.2%. After 3 and 5 years, 95.4% and 81.3% of patients respectively were stroke-free and 72.8% and 67.3% were alive. There were four recurrences and two of them related to stroke. Forty-nine(70%) stenoses operated on were symptomatic. Brain infarction was detected in 59.2% of patients who underwent computerized to mography scan or magnetic resonance imaging.

**CONCLUSIONS:**

Carotid endarterectomy was done in accordance with international standards. The most frequent cause of late death was myocardial infarction, and recurrences were related to stroke. Patients should be followed up closely.

## RESUMO

**CONTEXTO:**

A endarterectomia de carótida tem indicações e resultados definidos por estudos clínicos multicêntricos como o *European Carotid Surgery Trialists, North-American Symptomatic Carotid Endarterectomy Trial* e *Asymptomatic Carotid Atherosclerosis Study* . Os pacientes foram altamente selecionados, assim como os cirurgiões, para estes estudos. A prática clínica é diferente, porém os mesmos resultados devem ser atingidos.

**OBJETIVO:**

Estudar se as indicações, técnica e resultados imediatos e tardios da endarterectomia carotídea estão de acordo com os padrões definidos pelos estudos multicêntricos.

**TIPO DE ESTUDO:**

Estudo retrospectivo de relato de casuística.

**LOCAL:**

Hospital de cuidados terciários privado da cidade de São Paulo, Brasil.

**PARTICIPANTES:**

57 doentes foram submetidos a 70 endarterectomias de carótida durante período de 10 anos. A média de idades foi 66,4±7,8 anos; 43 (75,4%) eram homens, 41 (71,9%) hipertensos, 36 (63,1%) fumantes e 24 (21,0%) diabéticos. A estenose bilateral da carótida estava presente em 31 (54,3%) doentes, a oclusão arterial periférica em 32 (56,1%) e a cardiopatia isquêmica em 25 (43,1%). A arteriografia foi feita em todos os doentes e o mapeamento ultra-sonográfico dúplex em 41 (71,9%). Exames de imagem cerebral (tomografia computadorizada ou ressonância magnética) foram obtidos de 36 pacientes. O seguimento variou de um a 122 meses.

**VARIÁVEIS ESTUDADAS:**

Óbitos pósoperatórios imediatos e tardios, acidentes vasculares cerebrais isquêmicos pósoperatórios imediatos e tardios, recidiva da placa de ateroma e sintomas que definiram a indicação cirúrgica.

**RESULTADOS:**

Houve um óbito (1,4%) no período pós-operatório imediato causado por enfarte do miocárdio e dois acidentes vasculares cerebrais (2,8%) – taxa de complicações totais de 4,2%. 49 (70%) das estenoses operadas eram sintomáticas. Enfarte cerebral foi detectado 59,2% dos doentes submetidos a exames de imagem. Após três e cinco anos, 72,8% e 67,3% dos doentes respectivamente estão vivos e 95,4% e 81,3% estão livres de acidente vascular cerebral. Houve quatro recidivas do ateroma, dois relacionados a acidente vascular cerebral.

**CONCLUSÕES:**

A endarterectomia de carótida tem sido feita segundo os padrões internacionais, a causa de óbito tardio mais freqüente é o enfarte do miocárdio, as recidivas da placa estão relacionadas ao acidente vascular cerebral, o seguimento de pacientes operados deve ser rigoroso.

## INTRODUCTION

Cerebral ischemia has been a matter of controversy for centuries. Wepfer in 1658 described a case of a patient with right hemiplegia and occlusion of the left internal carotid artery, and related the carotid occlusion to the hemiplegia. Willis in 1665 described a case of internal carotid artery occlusion in an asymptomatic patient. He showed that the absence of symptoms was due to an anastomotic network communicating between the arteries irrigating the brain, which is now known as the circle of Willis. The idea that extracranial arterial lesions could cause cerebral symptoms was doubted.

At the beginning of the 20^th^ century it was demonstrated that carotid bifurcation stenosis can cause cerebral ischemia by the mechanism of clot embolism. Fifty years later the characteristics of the stenosing atheroma plaque at the carotid bifurcation were described and surgical correction proposed. In 1951 the first operation on a carotid stenosis was done by Carrea, Molins and Murphy in Argentina and in 1954, Eastcott, Pickering and Robb operated on an woman complaining of amaurosis fugax, thereby curing her.^1,2^

Carotid endarterectomy has become one of the most common operations done in the USA but some surgeons have questioned its efficacy.^3^ Some cooperative clinical trials have been done to establish the basis for indication of this surgical treatment. The European Carotid Surgery Trial studied symptomatic patients and showed that patients with 70% or more carotid stenosis have a lower risk of stroke when operated on.^4^ The North American Symptomatic Carotid Trial also showed that patients with 70% or more carotid stenosis benefited from surgery, and the benefit was greater the higher the degree of stenosis.^5^ The Veterans Administration Asymptomatic Trial studied patients whose stenosis was 50% or higher and demonstrated that patients who had had transient ischemic attacks or small cerebral infarction (non-incapacitating) did better after being operated on than did the nonoperated ones.^6^ The Asymptomatic Carotid Atherosclerosis Study showed that patients operated on with 60% or more stenosis had a lower risk of stroke after five years than patients on medical therapy.^7^

Although the advantages of carotid endarterectomy are well established, another important point should be emphasized: the training of the surgeon. If the immediate complication rate is high all the benefit to the population operated on is lost.^7^ In recent years some vascular surgery services have published the results of carotid endarterectomy, with the immediate morbidity plus mortality rate at a level of 2%.^8-10^ This is much lower than the predicted rate of 6% above which the benefits of the endarterectomy are lost.^5^ The recommendation of the Guidelines for Carotid Endarterectomy is that "the complication rate after carotid endarterectomy should be maintained at an extremely low rate (≤ 3%) by surgeons to keep the beneficial effects of carotid endarterectomy over medical therapy".^11^

The objective of this study was to examine whether our surgical practice was in accordance with the standards established by the multicentric trials, i.e. in terms of indications for surgery, technique, and immediate and late results.

## METHODS

A retrospective study was done reviewing records of 57 patients operated on consecutively by the same surgical team in a tertiary care private hospital in São Paulo, Brazil, from January 1987 to December 1997. Collaborators (AL, CH and GS) collected the data under the supervision of the senior author (ETA). All patients had been referred to this vascular surgery service by an internist or a cardiologist or neurologist. Risk factors such as diabetes, hypertension and smoking habits, contralateral carotid stenosis or obstruction, peripheral arterial and cardiac diseases and symptoms of cerebral ischemia were considered as co-variants. Presence of ischemic cerebral lesions detected by either Magnetic Resonance Imaging or Computerized Tomography, choice of surgical technique (use of shunts and patches) and type of anesthesia (general or regional) was also considered in the analyses. All patients had four-vessel angiography done by femoral catheterization before surgery, and the degree of stenosis was calculated based on this. Forty-one (71.9%) patients had also a duplex-scan of the neck arteries.

Clinical outcomes consisted of a combined end-point of early postoperative stroke and death rate, causes of late death, survival after endarterectomy, survival without stroke and survival without recurrence of atheroma plaque.

Patients were divided into groups according to age (above and below 70 years), sex, diabetes mellitus, symptoms (symptomatic vs. asymptomatic) relative to the stenosis operated on, internal carotid stump pressure and the use of shunt. Frequency of cervical nerve lesions, postoperative hypertensive crisis and bleeding was also studied. The postoperative strokes, both ipsilateral and contralateral, were diagnosed by clinical examination. Computerized tomography scan or magnetic resonance imaging was not utilized routinely in the postoperative period unless any clinical manifestation of stroke had appeared. All patients were referred back to their prior physicians during the 30-day postoperative period and information from this was taken into account.

All patients except one had a complete follow-up. They were examined after 30 days, three months, six months and then yearly. One patient was lost to follow-up 60 days after bilateral endarterectomy.

Carotid bifurcation duplex-scan was done after three months and then yearly. These examinations were always done at the same service that did the preoperative one, but not always by the same operator. Recurrence of atheroma plaque was defined as postoperative carotid stenosis above a degree of 50%.

At the end of follow-up, the senior author saw all patients that could still be seen. Information about late death and stroke was obtained from the family or attending physician when necessary.

Fisher's Exact Test was applied at a significance level of 5%. Survival curves were made according to the actuarial method and patients were included after the 30-day postoperative period. The SPSS for Windows program was used for the construction of tables and survival curves.

## RESULTS

Fifty-seven patients with the diagnosis of atherosclerotic carotid bifurcation stenosis were operated on. Forty-three were male (75.4%) and 14 (24.6%) female. Ages varied from 51 to 84 years (median of 66.4 ± 7.8 years). Forty-one (71.9%) were hypertensive, 36 (63.1%) current smokers and 24 (42.1%) had diabetes. Contralateral carotid occlusive disease was found in 32 (56.1%) patients and 6 (10.5%) of them presented total occlusion of the contralateral internal carotid artery. Ischemic heart disease was diagnosed in 25 (43.8%) patients, two of them with congestive heart failure. Peripheral arterial disease was detected in 29 (50.8%) patients.

There was no cerebrovascular event after angiography. Cerebral imaging made by computerized tomography scan or magnetic resonance imaging was obtained for 36 patients. These tests detected brain infarction in 20 (55.5%).

Seventy carotid endarterectomies were performed on these patients. Stenosis was between 50 and 70% in 5 cases and four of these were symptomatic.

Clinical manifestations of the carotid stenosis were transient ischemic attack (35 endarterectomies; 50.0%), minor or moderate stroke (14 endarterectomies; 20.0%). Eighteen (25.7%) carotid plaques were asymptomatic.

**Figure 1:**
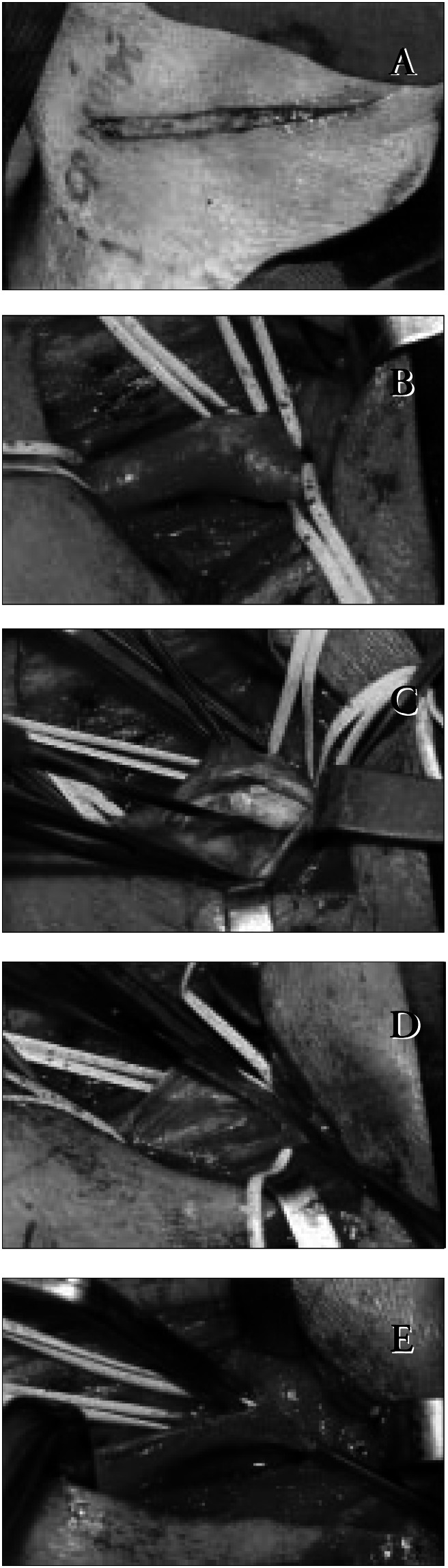
Carotid endarterectomy – Technical aspects.

Surgical technique varied little (Figure 1). After dissection of the carotid bifurcation, the patient was anticoagulated with 5000 m of intravenous heparin, and systolic blood pressure was elevated by 20 mmHg to a maximum of 160 mmHg. Common and external carotid arteries were clamped and internal carotid stump pressure was measured. If it was below 50 mmHg, a shunt was inserted: 14 (20%) shunts were required.

***A–Transverse incision. B–Carotid bifurcation dissected.***

***CeD–Endarterectomy technique. E–Arteriotomy suture.***

Stump pressure was below 50 mmHg on seven occasions but the shunt was not inserted due to technical difficulties: in six cases the stump pressure varied between 40 and 48 mmHg and in one case the pressure was 30 mmHg. Internal carotid artery kinking was corrected during endarterectomy three times: in these cases an eversion endarterectomy was performed after sectioning the origin of the internal carotid artery. A patch to close the arteriotomy was used four (5.7%) times. Carotid endarterectomy was associated with other operations in seven cases: femoral-tibial bypasses (3 cases), coronary artery bypass, hysterectomy, hip surgery and cholecystectomy (one case each).

General anesthesia was performed in 55 (78.5%) operations and regional anesthesia in 15 (21.5%). Two cases operated on under regional anesthesia fell into deep sleep (coma) after clamping and woke up immediately after insertion of the shunt; stump pressures were 23 and 38 mmHg.

The immediate results of the carotid endarterectomy were the following:

There was one death (1.4%) caused by myocardial infarction after a carotid endarterectomy associated to femoral-tibial bypass for critical ischemia in a diabetic patient. This patient had an occluded coronary artery bypass done 13 years earlier.

There were two patients with strokes (2.8%): one major with late sequelae and one minor with complete recovery. The patient with the major stroke had an internal carotid stump pressure of 30 mmHg and the shunt was not used. The other stroke occurred in a patient whose stump pressure was above 50 mmHg, but with a clamping time of over two hours due to technical problems and bleeding leading to hypotension during the clamping.

The complication (death + stroke) rate was 4.2%. There was no influence of age, sex, diabetes mellitus, symptoms, carotid stump pressure and the use of shunt on immediate results (Table 1). Postoperative hypertensive crises occurred after seven (10.0%) endarterectomies, and bleeding that required reoperation occurred after three (4.2%). Cranial nerve injuries occurred after 21 (32.3%) operations; nineteen were transient and two (2.8%) definitive.

**Table 1:** Distribution of post-operative death + stroke by age, sex, diabetes, symptoms, carotid stump pressure and the use of shunt after carotid endarterectomy

*Characteristics*	*No*	*Death + stroke*	*Yes*	*Total*	p value*
***Age, years***					
***<70***	***47***		***1***	***48***	
***>70***	***20***		***2***	***22***	***0.23***
***Sex***					
***Male***	***41***		***2***	***43***	
***Female***	***26***		***1***	***27***	***0.69***
***Diabetes***					
***No***	***33***		***1***	***34***	
***Yes***	***29***		***2***	***31***	***0.57***
***Symptoms***					
***No***	***21***		***0***	***21***	
***Yes***	***46***		***3***	***49***	***0.50***
***Carotid stump pressure***					
***>40 mmHg***	***55***		***2***	***67***	
***<12 mmHg***	***12***		***1***	***13***	***0.46***
***Shunt***					
***No***	***53***		***3***	***56***	
***Yes***	***14***		***0***	***14***	***0.50***

* Fisher's Exact test.

The late results were the following:

After a follow-up period of between one and 122 months (mean of 37.3), there were 15 deaths, seven of which were diabetic. Causes of death were myocardial infarction (8 cases), congestive heart failure (2 cases), chronic renal insufficiency (2 cases), lung cancer, immediate complications of aortic-femoral thrombo-endarterectomy, and stroke due to recurrence of atheroma plaque on carotid bifurcation after nine years (one case each).

**Figure 2:**
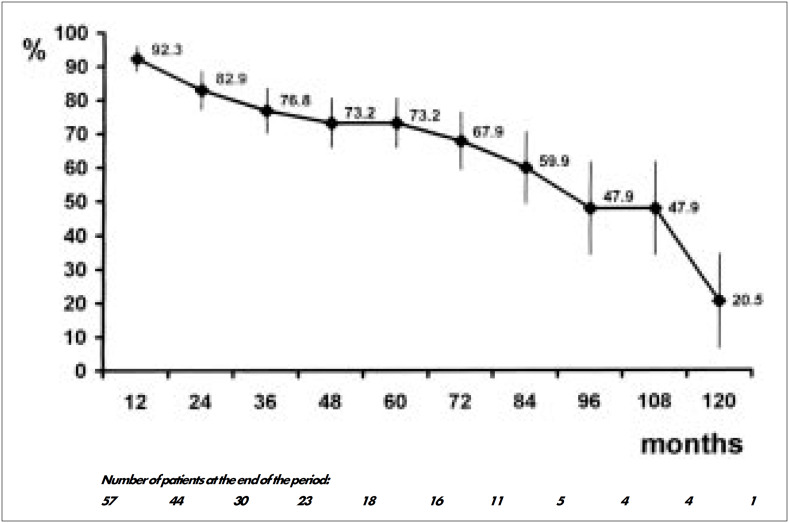
Survival of the population after carotid endarterectomy.

**Figure 3:**
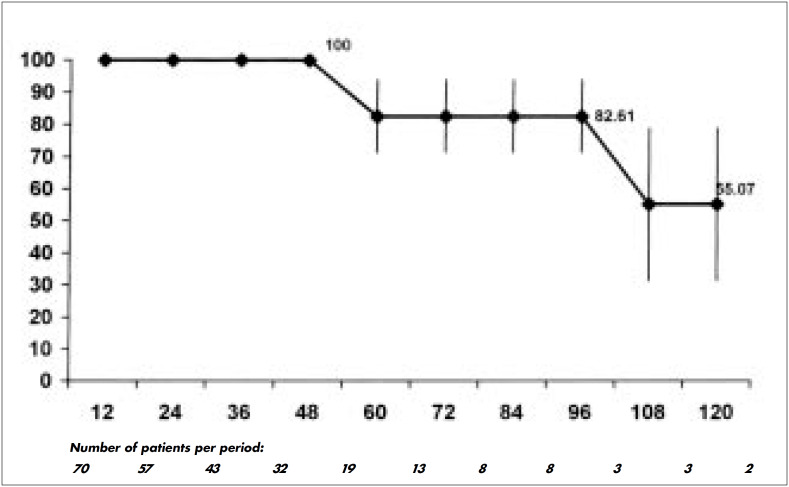
Rates of patients without stroke after carotid endarterectomy.

**Figure 4:**
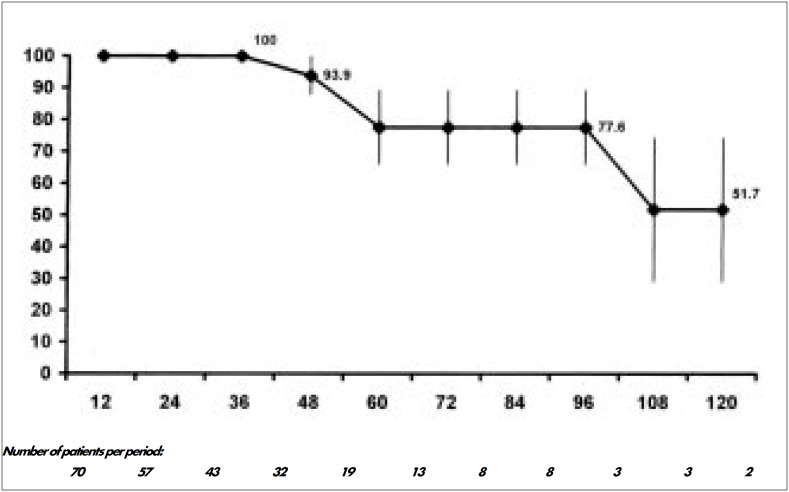
Rates of patients without recurrence of atheroma plaque after carotid endarterectomy.

As seen in Figure 2, 76.8 ± 6.5% and 73.2 ± 7.2% were alive after three and five years respectively. Also after three and five years, 100% and 82.6 ± 11.1% of patients respectively were stroke-free (Figure 3). Four (5.7%) recurrences of atheroma plaque were detected during followup. One occurred after 4 years, two after 5 years and one after 9 years. One patient had a bilateral recurrence, but was asymptomatic and refused to redo surgery. A second patient presented transient ischemic attacks and recurrence was diagnosed. He was reoperated and is doing well five years afterwards. A third patient's recurrence was followed by stroke and the patient died one month later. Figure 4 demonstrates that the recurrences were late in occurrence.

## DISCUSSION

This was a retrospective study and the same surgeon did all the operations. We think these kinds of studies are important because they show the clinical practice itself, since patients are not selected as in trials. The advantages of carotid endarterectomy have already been demonstrated in trials and we need to know whether we can obtain the same results in ordinary clinical practice. Our sample of patients showed little difference to that of the trials. We included patients older than 80 years, an age group that was excluded from the trials, as we think they should be operated on for stroke prevention. Our results demonstrated that there is no influence of age on immediate complication rate, as has also been demonstrated by other surgeons.^12-14^

The indication for operation followed the standards recommended by the multicentric trials. Most patients were symptomatic and the degree of stenosis was 70% or higher. There were five patients operated on with stenosis less than 70%. Although the Asymptomatic Carotic Atherosclerosis Study stated that stenosis above 60% should be operated, we believe that patients with borderline stenosis, especially symptomatic ones, should also be operated on if the immediate complication rate is low. Fortunately our borderline patients had no complications.

Another interesting point to note is the contralateral stenosis. It is commonly reported in the literature, but is mostly below 80%.^1^ Contralateral stenosis was present in 58.4% of our patients and total contralateral occlusion occurred in 11.3% of patients.

Peripheral arterial disease was associated with carotid stenosis in 52.8% of our patients. Such an association has not been emphasized in the literature. This high incidence of peripheral vascular disease is explicable, as it was the main reason patients sought out our service. Most of them came because of lower limb claudication or critical ischemia and the carotid stenosis was found during examination. Some of these patients had already had transient ischemic attacks or a non-incapacitating stroke but the carotid stenosis had not been treated (some neurologists in our country still do not believe in the advantages of surgical treatment for carotid stenosis). The presence of peripheral arterial disease demonstrates the severity of the atherosclerotic disease in these patients.

The degree of stenosis is established by duplex-scan in most services and many surgeons have operated on patients based only on this test.^15^ Arteriography is still necessary for indicating the procedure because it permits the study of whole-brain circulation and the diagnosis of other cerebrovascular diseases like aneurysms.

Cerebral imaging is an important test. Fifty-five percent of patients on which these tests were done had signs of brain infarction. It is important to know about any previous brain damage, especially if any neurological complications occur in the postoperative period. It has been demonstrated that silent brain infarcts occur in approximately 14% of asymptomatic patients and the incidence increases with the severity of symptoms.^16^ The influence of these lesions on outcome is still unclear but there is evidence that the presence of an infarct on computerized tomography is associated with higher risk of stroke.^17^

The surgical technique used was standardized and not different from that used by most surgeons. Bilateral stenoses were operated on one after the other with an interval of one to two weeks. This time is not well established: some surgeons wait 15 days to operate on contralateral stenosis, some wait two days and there are surgeons doing both endarterectomies at the same time.^18^ Most surgeons prefer to do bilateral endarterectomy with an interval of time between them.

Shunt use was selective and based on data obtained from internal carotid stump pressure. There are other ways to detect brain troubles during clamping: Plestis et al (1997) demonstrated a remarkable reduction in the postoperative complication rate by using continuous electroencephalographic monitoring and selective shunting.^19^ Some surgeons never use shunts while others use it routinely.^9,20,21^ Most surgeons use shunts selectively.

Internal carotid stump pressure measurements are easy to perform, costeffective and can be used in any hospital as a way of detecting the increased risk of stroke during clamping.^22^ Two of our patients went into coma at the moment of clamping and had carotid stump pressures below 30 mmHg. Both woke up immediately after shunting.

Most of our patients were operated on under general anesthesia, as in many other services.^9,10^ Some vascular surgeons believe that operating on a patient under regional anesthesia is the best way to detect any brain problem during clamping, and they do it routinely.^23^ Up until now, no difference has been demonstrated between general or regional anesthesia.^24^

The mortality rate of this series was 1.4%. The only death occurred in a diabetic patient, with gangrene of the big toe and 90% asymptomatic carotid stenosis. Thirteen years earlier, this patient had undergone myocardial revascularization. The bypasses had already occluded and it was not feasible to redo the operation. This patient died of a myocardial infarction immediately after carotid endarterectomy done in association with a femoral-tibial bypass. The mortality rate for carotid endarterectomy done in association with other operations is not often shown in the literature: some studies show that it is higher. Among this patient sample, there was no death after carotid endarterectomy that was not associated with other operations.

Patients over 80 years of age were included. Some papers have demonstrated that it is possible to operate on patients at this age with same mortality rates as on patients under 80 years and that the benefit is long-lasting.^13,14^

Our immediate death + stroke complication rate was 4.3%, which is below the maximum accepted after carotid endarterectomies (6%) and close to that recommended by the Guidelines for Carotid Endarterectomy of the Special Writing Group of the Stroke Council, American Heart Association.^5,11^ If we consider one death plus one definitive sequelae (one patient that had a postoperative stroke recovered completely), we obtain a death + stroke rate of 2.8%. This is very close to that obtained by others, and is at the level recommended by the Guidelines.^9-11,25^ In this series there was no influence of age, sex, diabetes mellitus, symptoms, carotid artery back pressure and the use of shunts on immediate results. There were no complications after operations on asymptomatic patients. We agree with Kucey et al. (1998) that, although we were studying a small number of patients, the most important factor in preventing complications is the experience of the surgeon and anesthesiologist.^26^ One of the postoperative strokes happened during a difficult operation with bleeding and hypotension during clamping. The other one may be explained by embolization during dissection.

Other complications are the postoperative hypertensive crises and injury to cranial nerves. Occurrence of both complications diminishes as the experience of the surgical team and anesthesiologist increases. Most injuries to nervous trunks are reversible, but may be definitive and disabling.^27^ When a transverse skin incision is used, the most frequent injury is to the great auricular nerve.^12^

Late results revealed that after 3 and 5 years, 73.2% and 67.9% of patients, respectively, were alive and the commonest cause of late death was myocardial infarction. These data do not differ from those published in the international literature.

After the same periods of time, 100% and 82.6% of patients, respectively, were strokefree. These data are also similar to those of the multicentric trials and to those published recently by Hallet et al. (1998).^25^

The recurrence rate has been studied in many papers and some surgeons believe that closure of the arteriotomy with patches prevents the recurrence of atheroma plaque.^28^^,^^29^ In the USA about 16% of patients have their arteriotomy closed by a patch.^30^ Salles et al. (1998) found that closing the arteriotomy with patches increases clamping time and also the immediate complication rate.^1^ In our series, the recurrence rate was 6.1% and all of them occurred after 4.5 years, with half of them being symptomatic. The literature shows recurrence rates for symptomatic plaque varying from 2% to 4% after 5 years. The use of patches is not well established yet.^31,32^ We think the selective use of patches in small-caliber arteries, as done in this series, is adequate.

Finally, we should remember that the small number of patients in this sample is a limitation. All differences between the groups may have been underestimated and the results should be viewed with care. Another limitation is that postoperative stroke was diagnosed clinically and the silent strokes were not detected because computerized tomography or magnetic resonance imaging was not routinely done, either immediately or later. The technical aspects of carotid endarterectomy are still under discussion by the principal surgical services around the world.

## CONCLUSION

Carotid endarterectomy was done in accordance with the principles recommended by the trials. The most frequent cause of immediate and late death is myocardial infarction, and atheroma plaque recurrence is related to stroke. The patient should be followed up closely, making periodic duplexscans of the operated carotid artery.
